# Microbial BMAA elicits mitochondrial dysfunction, innate immunity activation, and Alzheimer’s disease features in cortical neurons

**DOI:** 10.1186/s12974-020-02004-y

**Published:** 2020-11-05

**Authors:** Diana F. Silva, Emanuel Candeias, A. Raquel Esteves, João D. Magalhães, I. Luísa Ferreira, Daniela Nunes-Costa, A. Cristina Rego, Nuno Empadinhas, Sandra M. Cardoso

**Affiliations:** 1 CNC–Center for Neuroscience and Cell Biology, University of Coimbra, Largo Marquês de Pombal, 3004-517 Coimbra, Portugal; 2 IIIUC-Institute for Interdisciplinary Research, University of Coimbra, Coimbra, Portugal; 3 Ph.D. Programme in Biomedicine and Experimental Biology (PDBEB), Institute for Interdisciplinary Research, University of Coimbra, Coimbra, Portugal; 4 Institute of Biochemistry, Faculty of Medicine, University of Coimbra, Coimbra, Portugal; 5 Institute of Cellular and Molecular Biology, Faculty of Medicine, University of Coimbra, Coimbra, Portugal

**Keywords:** Alzheimer’s disease, β-*N*-Methylamino-l-alanine, Mitochondrial dysfunction, Neuronal innate immunity, Neurodegeneration

## Abstract

**Background:**

After decades of research recognizing it as a complex multifactorial disorder, sporadic Alzheimer’s disease (sAD) still has no known etiology. Adding to the myriad of different pathways involved, bacterial neurotoxins are assuming greater importance in the etiology and/or progression of sAD. β-*N*-Methylamino-l-alanine (BMAA), a neurotoxin produced by some microorganisms namely cyanobacteria, was previously detected in the brains of AD patients. Indeed, the consumption of BMAA-enriched foods has been proposed to induce amyotrophic lateral sclerosis-parkinsonism-dementia complex (ALS-PDC), which implicated this microbial metabolite in neurodegeneration mechanisms.

**Methods:**

Freshly isolated mitochondria from C57BL/6 mice were treated with BMAA and O_2_ consumption rates were determined. O_2_ consumption and glycolysis rates were also measured in mouse primary cortical neuronal cultures. Further, mitochondrial membrane potential and ROS production were evaluated by fluorimetry and the integrity of mitochondrial network was examined by immunofluorescence. Finally, the ability of BMAA to activate neuronal innate immunity was quantified by addressing TLRs (Toll-like receptors) expression, p65 NF-κB translocation into the nucleus, increased expression of NLRP3 (Nod-like receptor 3), and pro-IL-1β. Caspase-1 activity was evaluated using a colorimetric substrate and mature IL-1β levels were also determined by ELISA.

**Results:**

Treatment with BMAA reduced O_2_ consumption rates in both isolated mitochondria and in primary cortical cultures, with additional reduced glycolytic rates, decrease mitochondrial potential and increased ROS production. The mitochondrial network was found to be fragmented, which resulted in cardiolipin exposure that stimulated inflammasome NLRP3, reinforced by decreased mitochondrial turnover, as indicated by increased p62 levels. BMAA treatment also activated neuronal extracellular TLR4 and intracellular TLR3, inducing p65 NF-κB translocation into the nucleus and activating the transcription of NLRP3 and pro-IL-1β. Increased caspase-1 activity resulted in elevated levels of mature IL-1β. These alterations in mitochondrial metabolism and inflammation increased Tau phosphorylation and Aβ peptides production, two hallmarks of AD.

**Conclusions:**

Here we propose a unifying mechanism for AD neurodegeneration in which a microbial toxin can induce mitochondrial dysfunction and activate neuronal innate immunity, which ultimately results in Tau and Aβ pathology. Our data show that neurons, alone, can mount inflammatory responses, a role previously attributed exclusively to glial cells.

**Supplementary Information:**

The online version contains supplementary material available at 10.1186/s12974-020-02004-y.

## Background

Alzheimer’s disease (AD), a progressive neurodegenerative disorder associated with memory deficits and cognitive decline, is characterized by the presence of amyloid plaques and neurofibrillary tangles in susceptible brain areas [1]. One of the main initial alterations observed in the brains of AD patients is regional hypometabolism, which implicates changes in neuronal energy metabolic pathways. Although the primary insult in AD is still a subject of debate, the observed brain metabolic abnormalities are thought to mirror impaired mitochondrial function [2]. Indeed, it has been described that mitochondrial metabolism and dynamics are affected not only in the cerebral cortex and hippocampus but also in peripheral cells, namely platelets, fibroblasts, and lymphocytes of AD patients [3]. Additionally, neuroinflammation, which consists in the imbalance between anti-inflammatory and pro-inflammatory signaling, is a key event in the AD neurodegenerative process resulting in the activation of microglia cells and in the release of various cytokines that contribute to neuronal damage [4]. Increased levels of pro-inflammatory markers were detected in the CSF of AD patients, namely IL-8, IL-6, and TREM2 [5, 6], as well as in brain tissue where the activation of microglial cells was assessed by examining CD68 and Iba1 positive cells [7]. While microglial cells are considered to be responsible for the majority of the inflammatory responses in the central nervous system (CNS), neurons can also mount innate immune responses. It was previously described that neurons may activate innate immunity in response to pathogen infection or to danger associated molecular patterns (DAMPs) [8, 9]. Therefore, upon a given damage, mitochondria will present increased network fragmentation [10] expose DAMPs [11], which may be enough to activate neuronal innate immunity, with cytokine production to recruit and activate local microglia. The etiology of sporadic AD has been addressed by several different hypotheses such as the mitochondrial cascade hypothesis [12], the amyloid cascade hypothesis [13], the neuroinflammation hypothesis [14], and the infectious theory [15]. Indeed, these different hypotheses are complementary instead of mutually exclusive and lead us to propose a unifying theory for the onset of AD involving bacterial toxins. In this, certain microbial toxins from dietary sources or hypothetically produced by gut microbiota may inadvertently target neuronal mitochondria as a consequence of their evolution from ancestral bacteria by endosymbiosis [16], which will activate neuronal innate immunity, triggering neuroinflammation [17].

Mounting evidence suggests that the neurotoxin β-*N*-methylamino-l-alanine (BMAA), a natural non-proteinogenic diamino acid produced by cyanobacteria and by some other microbes, biomagnifies through the food chain in some ecosystems [18]. Since it accumulates for example in seafood such as shellfish, prawns, and fish, BMAA could, in addition to host genetic factors, represent an environmental trigger for the onset of neurodegeneration [19]. BMAA was initially discovered in the seeds of cycad plants, whose consumption was considered to be the cause of the amyotrophic lateral sclerosis-parkinsonism dementia complex (ALS-PDC) [20, 21] that affects the habitants from the Pacific island of Guam, from the Kii peninsula of Japan, and from western New Guinea [22]. BMAA was also found in the brains of two Canadian AD patients [21], which suggested it could be involved in AD etiology. To date, BMAA is known to cross the blood-brain barrier (BBB) [23] and exert neurotoxicity through competitive binding to various glutamate receptors namely NMDA and AMPA receptors [24]. BMAA activates glutamate receptors and shifts cellular ionic enrichment resulting in an increase in Na^+^ [25] and Ca^2+^ [26] and decrease in K^+^ concentrations [25]. Increased Ca^2+^ disrupts normal mitochondrial function leading to ROS overproduction and cytochrome *c* release into the cytosol, which initiates apoptosis [27, 28]. Furthermore, there is evidence that BMAA is transported into neurons and astrocytes by the cystine/glutamate antiporter (system x_c_-) enabling it to also affect these cells through its action on intracellular targets [29]. Indeed, accumulating evidence suggests that BMAA may cause protein misfolding due to its misincorporation (in place of serine) into their primary structure or through strong electrostatic interactions with nascent chains, which may result in protein aggregation, ER stress, and apoptosis [19, 30, 31], compatible with progressive neurodegeneration. Other intracellular effects of BMAA have been proposed to contribute to its neurotoxicity, including inhibition of catalase activity and strong association with melanin and neuromelanin [28, 32].

Here we propose a novel molecular pathway for BMAA-dependent neuronal damage which unifies key aspects known to mediate AD pathology: mitochondrial dysfunction, neuronal inflammation, Tau hyperphosphorylation, and Aβ oligomers accumulation. We challenge the current mainstream understanding on CNS inflammation which postulates that an initial activation of glial cells with concomitant production of cytokines will ultimately damage neurons [33]. Furthermore, we present evidence that neurons alone can mount inflammatory responses with the activation of neuronal innate immunity through Nod-like receptor (NLRP3) also called inflammasome. BMAA is toxic to mitochondria as it activates intracellular Toll-like receptors (TLRs), the translocation of p65 NF-κB into the nucleus that induces the expression of pro-IL-1β and NLRP3 inflammasome components. We propose that some bacterial toxins, such as BMAA, are able to impair mitochondrial function and thus activate neuronal innate immunity. Cytokines released will then further activate glia cells building the inflammatory response that characterizes AD pathology.

## Methods

**Table:** 

Chemicals	Manufacturer
10-*N*-Nonyl acridine orange (NAO)	Enzo Lifesciences (Lausen, Switzerland); Cat. No. 08091739
5-Fluoro-2’-deoxyuridine (FDU)	Sigma Chemical Co (St. Louis, MO, USA); Cat. No. F0503
2-Deoxyglucose	Sigma Chemical Co (St. Louis, MO, USA); Cat. No. D8375
Ammonium chloride (NH_4_Cl)	Sigma Chemical Co (St. Louis, MO, USA); Cat. No. 9434
S(+)-2-Amino-3-(methylamino) propionic acid hydrochloride (BMAA)	iChemical CO., LTD Shanghai; Cat. No. EBD13091
Antimycin	Sigma Chemical Co (St. Louis, MO, USA); Cat. No. A8674
Calcium Green 5N	Invitrogen, Molecular Probes, Life Technologies, (Eugene, OR, USA); Cat. No. C3739
Carbonyl cyanide 4-(trifluoromethoxy)phenylhydrazone (FCCP)	Sigma Chemical Co (St. Louis, MO, USA); Cat. No. C2920
Carbonyl cyanide m-chlorophenyl hydrazone (CCCP)	Sigma Chemical Co (St. Louis, MO, USA); Cat. No. C2759
Caspase 1 substrate (Ac-VAD-4-methoxy-2-naphtylamide)	Sigma Chemical Co (St. Louis, MO, USA); Cat. No. SCP0066
3-(4,5-Dimethylthiazol-2-yl) 2,5-diphenyltetrazolium bromide (MTT)	Sigma Chemical Co (St. Louis, MO, USA); Cat. No. M2128
Leupeptin	Sigma Chemical Co (St. Louis, MO, USA); Cat. No. L2023
Lipopolysaccharides (LPS)	026: B6; Sigma Chemical Co (St. Louis, MO, USA; Cat. No. L2654
MitoPY1	Sigma Chemical Co (St. Louis, MO, USA); Cat. No. SML0734
Oligomycin	Alfa Aesar (Karlsruhe, Germany); Cat. No. J60211
Poly(ethyleneimine) (PEI)	Sigma Chemical Co (St. Louis, MO, USA); Cat. No. 408700
Rotenone	Sigma Chemical Co (St. Louis, MO, USA); Cat. No. R8875
Tetramethylrhodamine, methyl ester (TMRM)	Molecular Probes (Eugene, OR, USA); Cat. No. T668

### BMAA preparation and storage

BMAA (98% purity) obtained from iChemical CO (LTD, Shanghai) was weighed and dissolved in sterile deionized water to obtain a stock solution of 970 mM. From this master stock, a working stock was prepared by diluting BMAA in sterile water at 60 mM. Both master stock and the working stock were aliquoted to avoid freeze and thaw cycles and stored at − 20 °C.

### Isolation and treatment of primary neuronal cultures

Primary neuronal cultures were obtained as described in [34], with minor modifications. Frontal cortices removed from embryonic days 15–16 of C57BL/6 mice were aseptically dissected and combined in Ca^2+^ and Mg^2+^ free Krebs buffer [120 mM NaCl, 4.8 mM KCl, 1.2 mM KH_2_PO4, 13 mM glucose, 10 mM HEPES (pH 7.4)] and then incubated in Krebs solution supplemented with BSA (0.3 g/L) containing trypsin (0.5 g/L) for 10 min at 37 °C. Tissue digestion was stopped by the addition of trypsin inhibitor (type II-S; 0.75 g/L) in Krebs buffer, followed by a centrifugation at 140×*g* for 5 min. After washing the pellet once with Krebs buffer, the cells were dissociated mechanically and resuspended in fresh Neurobasal medium (Merk Life Science SLU, from Sigma St. Louis, MO, USA; Cat. No. 1.00289.0100), supplemented with 0.2 mM l-glutamine (Sigma St. Louis, MO, USA; Cat. No. G3126), 2% B-27 (Gibco, ThermoFisher Scientific, Waltham, MA, USA; Cat. No. 17504-044) supplement, penicillin (100,000 U/L), and streptomycin (100 mg/L). The cells were seeded on poly-l-lysine (Sigma St. Louis, MO, USA; Cat. No. P1399) (0.1 g/L)-coated dishes at a density of 0.33 × 10^6^ cells/cm^2^. For western blot analysis, neurons were plated in 6-well plates; for mitochondrial membrane potential and mitochondrial ROS assays, neurons were plated in 48-well plates; for Seahorse XF24 apparatus measurements, the manufacturer’s plates were coated with poly-l-lysine prior to cell seeding. The day after seeding, cultures were treated with 5 μM FDU to prevent glial cell proliferation. The presence of glial cells in our cultures was assessed after 6 days in culture, with or without FDU. We immunolabeled our cultures with the neuronal marker microtubule-associated protein 2 (MAP2; red) and with the astrocytic marker glial fibrillary acidic protein (GFAP; green) or the ionized calcium-binding adaptor molecule 1 (Iba1; green). Our results showed virtually undetectable astrocytes and microglial cells as evidenced by a lack of green staining in Fig. S1a2 and a3 (Fig. S1). The cultures were maintained in serum-free Neurobasal medium (Gibco, ThermoFisher Scientific, Waltham, MA, USA; Cat. No. 21103-049) supplemented with B-27 at 37 °C in a humidified atmosphere of 5% CO_2_, 95% air for 6 days before treatment, to allow neuronal differentiation. After 6 days in vitro, cultured neurons were treated with 3 mM BMAA, 1 μg/mL LPS, and 1 μM CCCP, for 48 h before fixation or harvesting. Where indicated, 20 mM NH_4_Cl and 20 mM leupeptin were added in the culture medium in the last 4 h of the treatment to monitor autophagy. Also, for BMAA time-course experiments on innate immunity activation, 3 mM BMAA were added to cortical neurons for 6, 24, and 48 h.

### SHSY-5Y cell maintenance and treatments

Human neuroblastoma SH-SY5Y cell line (ATCC-CRL-2266) was grown in 75-cm^2^ tissue flasks in DMEM and Ham’s F12 (Gibco, ThermoFisher Scientific, Waltham, MA, USA; Cat. No. 42400-028) medium with 10% supplemental non-dialyzed fetal bovine serum and 100 IU/ml penicillin and 50 μg/ml streptomycin. Cells were maintained at 37 °C in a humidified incubator under an atmosphere of 95% air and 5% CO_2_. Approximately 50,000 cells/well were plated in 48-well plates prior to the TMRM assay. Medium was refreshed before incubation with 3 mM BMAA for 48 h.

### Fresh mitochondria isolation from mouse cortical tissue and treatments

Pre-frontal cortex tissue from C57BL/6 mice (adult females) was washed in ice-cold isolation buffer containing 225 mM mannitol, 75 mM sucrose, 1 mM EGTA, 5 mM HEPES, and pH 7.2/KOH. Cortical mitochondria were then isolated using discontinuous Percoll density gradient centrifugation, according to Ferreira and collaborators with some minor modifications [35]. For this purpose, pre-frontal cortical tissues were homogenized with 25 up and down strokes in Dounce All-Glass Tissue Grinder (Kontes Glass Co., Vineland, NJ, USA) using pestle A (clearance, 0.07–0.12 mm) followed by 25 up and down strokes with pestle B (clearance, 0.02–0.056 mm). After a brief centrifugation at 1100×*g* for 2 min at 4 °C, the supernatant was mixed with freshly made 80% Percoll prepared in 1 M sucrose, 50 mM HEPES, 10 mM EGTA, and pH 7.0, then carefully layered on the top of freshly made 10% Percoll (prepared from 80% Percoll) and further centrifuged at 18500×*g* for 10 min at 4 °C. Supernatant was discarded including the cloudy myelin containing fraction leaving the mitochondria-enriched pellet in the bottom of the tube and the pellet resuspended in 1 mL of washing buffer containing 250 mM sucrose, 5 mM HEPES-KOH, 0.1 mM EGTA, and pH 7.2 and centrifuged again at 10,000×*g* for 5 min at 4 °C. Finally, the mitochondrial pellet was resuspended in ice-cold washing buffer and the amount of protein quantified by the Bio-Rad protein assay (BioRad, CA, USA; Cat. No. 500-0006). Mitochondria (5 μg) were resuspended in washing buffer and loaded in each well. Isolated mitochondria were kept on ice until use on Seahorse apparatus in an assay based upon fluorimetric detection of O_2_ and H^+^ levels [35, 36]. Isolated mitochondria were solubilized in ice-cold mitochondrial assay solution (MAS) containing 70 mM sucrose, 220 mM mannitol, 10 mM KH_2_PO_4_, 5 mM MgCl_2_, 2 mM HEPES, 1 mM EGTA, pH 7.2, plus 10 mM succinate, and 2 μM rotenone substrates. The multiwell plate pre-coated with polyethyleneimine (PEI, 1:15,000 dilution from a 50% solution) containing mitochondria was then centrifuged at 2200×*g* for 20 min at 4 °C. In order to evaluate the mitochondria attachment efficiency, the plates were visualized under light microscopy using 20× magnification to ensure consistent adherence to the wells (data not shown). For Ca^2+^ uptake capacity measurements, mitochondria were incubated in medium containing 125 mM KCl, 0.5 mM MgCl_2_, 3 mM KH_2_PO_4_, 10 mM Hepes, pH 7.4, and 10 μM EGTA, supplemented either with 3 mM pyruvate, 1 mM malate, 3 mM succinate, 3 mM glutamate, 0.1 mM ADP, or 1 μM oligomycin. Then, mitochondria were incubated for 30 min at 30 °C in the absence or in the presence of 0.5 mM, 1 mM, and 3 mM of BMAA.

### Mitochondrial function

#### Mitochondrial Ca^2+^ uptake capacity

Mitochondrial Ca^2+^ uptake was measured fluorometrically in the presence of the Ca^2+^-sensitive fluorescent dye Calcium Green 5N (Invitrogen, Molecular Probes, Life Technologies, Eugene, OR, USA; Cat. No. C3739), using excitation and emission wavelengths of 506 nm and 532 nm, respectively, according [37] with some minor modifications. Ca^2+^ uptake was evaluated using a Spectramax Plus 384 spectrophotometer (Molecular Devices, Sunnyvale, CA, USA). Calcium Green is a cell-impermeant visible light-excitable Ca^2+^ indicator that exhibits an increase in fluorescence emission intensity upon binding to Ca^2+^; thus, a decrease in the Calcium Green fluorescence is function of external Ca^2+^ concentration that indicates the capacity of mitochondria to handle Ca^2+^. Briefly, 5 μg cortical isolated mitochondria were added to the standard incubation medium contained 125 mM KCl, 0.5 mM MgCl_2_, 3 mM KH_2_PO_4_, 10 mM Hepes, pH 7.4, 10 μM EGTA, supplemented either with 3 mM pyruvate, 1 mM malate, 3 mM succinate, 3 mM glutamate, 0.1 mM ADP, and 1 μM oligomycin plus 150 nM Calcium Green 5N. After a basal fluorescence record, four pulses of 10 μM CaCl_2_ pulse were added and fluorescence measured during 10 min. When desired, four pulses of 10 μM CaCl_2_ were added every 4 min and calcium handling capacity plotted as a decrease in fluorescence units (RFU), which reflects the rate of decrease of Calcium Green-5N fluorescence.

#### Seahorse XF24 extracellular flux analyzer measurements

##### In isolated mitochondria

After the treatments with 0.5, 1, and 3 mM BMAA described in the “Fresh mitochondria isolation from mouse cortical tissue and treatments” section, oxygen consumption rate (OCR) was measured using Seahorse XF24 extracellular flux analyzer (Agilent, Santa Clara, CA, USA). After an incubation period of 8 min at 37 °C in CO_2_-free incubator, the 24-well plate was transferred to the Seahorse XF24 flux analyzer. OCR respiration measurements were made using a 1-min mix, 30-s wait, and 3-min read cycling protocol. The levels of respiratory coupling were analyzed in MAS containing succinate (10 mM; Cx II substrate) plus rotenone (2 mM; Cx I inhibitor). Mitochondria were then energized by adding ADP (4 mM); respiration driven by ATP synthesis was then prevented by addition of oligomycin (2.5 μM; inhibitor of ATP synthase). The addition of the uncoupler FCCP (4 μM) caused an increase in OCR reflecting the maximal respiratory chain activity as well as the maximal substrate oxidation rate; finally, antimycin A (4 μM; Cx III inhibitor) was added to fully block the respiratory chain and the residual OCR.

##### In cortical neurons

Neuronal mitochondria OCR was evaluated by seeding approximately 100,000 cells per well in the 24-well cell culture microplates provided by the manufacturer (Agilent, Santa Clara, CA, USA; Cat. No. 100777-004). After neuronal differentiation and treatments 1 h before placing culture microplates in the Seahorse analyzer, the cells were washed in unbuffered DMEM from Sigma Chemical Co (St. Louis, MO, USA; Cat. No. 5030) without HEPES and sodium bicarbonate; the medium was then changed to the actual assay medium, which consisted of unbuffered DMEM containing 25 mM glucose, 1.82 mM sodium pyruvate, and 0.2 mM L-glutamine, at pH 7.4. OCR respiration measurements were made using a 1-min mix, 30-s wait, and 3-min read cycling protocol. During the first 3 reading periods, basal neuronal OCRs were determined. After the third reading, wells were injected with 1 μM of oligomycin and the resulting OCR was measured over 3 reading cycles. After this, two injections of 2 μM CCCP were performed and for each injection OCR was recorded over 3 cycles. Finally, a mixture of rotenone (2 μM) and antimycin A (2 μM) measured the resultant OCR over 3 reading cycles.

For glycolysis analysis on the assay day, neurons were washed in unbuffered DMEM followed by placement of the cells in unbuffered DMEM supplemented with 0.2 mM l-glutamine and 1.82 mM sodium pyruvate without glucose or bovine serum albumin (BSA). The microculture plates were then placed in a non-CO_2_ incubator at 37 °C for 1 h before the assay was performed in the Seahorse analyzer. Extracellular acidification rate (ECAR) measurements were made using a 1-min mix, 30-s wait, and 3-min read cycling protocol. After baseline measurements of 3 reading cycles, glucose was added to each well at a concentration of 10 mM and another 3 cycles were measured. Next, oligomycin was injected to each well (final concentration of 1 μM) and 3 readings were recorded. Finally, an injection of 2-deoxyglucose (final concentration of 100 mM) provides a non-glycolysis ECAR.

#### Mitochondrial membrane potential (ΔΨmit) analysis

Mitochondrial membrane potential (ΔΨmit) was detected using the fluorescent dye TMRM. After the described treatments, SHSY-5Y cells and cortical neurons were loaded with 300 nM of TMRM (in the dark, at 37 °C) for 1 h. The fluorescence of TMRM (λexc = 540 nm; λem = 590 nm) was recorded using a Spectramax Plus 384 spectrofluorometer (Molecular Devices) during 5 min before (base line) and 3 min after mitochondrial depolarization with CCCP. Maximal mitochondrial depolarization (Δψm collapse) was performed by adding 1 μM CCCP (proton ionophore), which was always preceded by oligomycin (2 μg/ml) to prevent ATP synthase reversal. The dye retention was determined by the difference between maximum fluorescence (after depolarization) and the basal value of fluorescence and expressed in relation to untreated condition. Since TMRM is a cell-permeant cationic dye, it is readily sequestered by functional mitochondria and a decrease of cellular retention of these dyes has been associated with a decrease in Δψm.

#### Mitochondrial reactive oxygen species measurements

MitoPY1 is a fluorescent mitochondrial hydrogen peroxide (H_2_O_2_) indicator and was used to determine H_2_O_2_ concentration in mitochondria of living cortical neurons. Briefly, after the treatments described, neurons were incubated in 300 nM of MitoPY1 dye in Krebs medium for 1 h at 37 °C. Fluorescence measurements were taken during 5 min (λexc = 503 nm; λem = 540 nm) as basal readings. Afterwards, neurons were challenged with 5 μM of rotenone (complex I inhibitor) to evaluate the susceptibility of mitochondria, and fluorescence measurements were taken for 30 min (λexc = 503 nm; λem = 540 nm). The values were obtained as a subtraction of higher value after rotenone was added minus baseline readings and then expressed in relation to untreated condition.

### 10-*N*-Nonyl acridine orange (NAO) staining

10-*N*-Nonyl acridine orange (NAO) binds to negatively charged phospholipids cardiolipin, phosphatidylinositol, and phosphatidylserine, but with higher affinity to cardiolipin, and is largely independent of mitochondrial membrane potential. Cardiolipin distribution and fluorescence was measured using the NAO probe. After treatments, neurons were washed with HBSS (5.36 mM KCl, 0.44 mM KH_2_PO_4_, 137 mM NaCl, 4.16 mM NaHCO_3_, 0.34 mM NaH_2_PO_4_.H_2_O, 5 mM Glucose, 5.36 mM Sodium Pyruvate, 5.36 mM Hepes, pH 7.2) and then loaded in the dark with 100 nM Cardiolipin in HBSS for 1 h. After a gentle wash, cells were kept in HBSS during image acquisition. Images were obtained using a Plan-Apochromat/1.4NA 63× lens on an Axio Observer.Z1 confocal microscope (Zeiss Microscopy, Germany) with Zeiss LSM 710 software.

The images of cells stained with NAO were extracted to grayscale, inverted to show NAO-specific fluorescence as black pixels and thresholded to optimally resolve NAO staining. Background fluorescence and specific NAO fluorescence were determined. The final value for fluorescence intensity resulted from the subtraction of background fluorescence from specific fluorescence and the result was further divided by the number of cells from each acquired image.

### MTT cell viability assay

Neuronal viability was determined by the colorimetric MTT (3-(4,5-dimethylthiazol-2-yl) 2,5-diphenyltetrazolium bromide) assay. In viable cells, NAD(P)H-dependent oxidoreductase enzymes reduce the MTT reagent to formazan, an insoluble crystalline product with a purple color that absorbs light at 570 nm. Following neuronal treatment with increasing concentrations of BMAA (250 μM, 500 μM, 1 mM and 3 mM) for 48 h, 0.5 mg/ml of MTT was added to each well. Neurons were then incubated at 37 °C for 2 h. At the end of the incubation period, the formazan precipitates were solubilized with 0.5 ml of acidic isopropanol (0.04 M HCl/Isopropanol). The absorbance was measured at 570 nm.

### Immunostaining

For immunostaining assays, approximately 100,000 cells per well were seeded and neuronal cultures were differentiated on poly-l-lysine (Sigma St. Louis, MO, USA; Cat. No. P1399) (0.1 g/L)-coated ibidi® μ-Slide eight-well plates from GmbH (Germany) (Cat. No. 80821). Ibidi® plates are microscopy slides where neurons are imaged on a No. 1.5 polymer coverslip bottom suitable for most microscopy techniques. Following the differentiation and cell treatments, cultures were fixed for 10 min at room temperature using 4% paraformaldehyde supplemented with 4% sucrose. Fixed cells were washed again with PBS (137 mM NaCl, 2.7 mM KCl, 10 mM Na_2_HPO_4_, 1.8 mM KH_2_PO_4_) (Sigma Chemical Co (St. Louis, MO, USA) Cat. No. D8537), permeabilized with 0.2% Triton X-100 during 3 min, and blocked with 3% BSA for 30 min. The permeabilized cells were incubated with primary antibodies: 1:200 anti-TOM20 (Cat. No. sc-11415) and 1:200 anti-GFAP (Cat. No. sc-71143) both from Santa Cruz Biotechnology (Santa Cruz, CA, USA); 1:200 anti-MAP2 from Abcam (Cambridge, UK; Cat. No. ab5392); and 1:500 anti-Iba1 from FUJIFILM Wako Chemicals USA Corporation (Cat. No. 019-19741), overnight in a wet chamber, at 4 °C. Subsequently, cells were incubated 1 h with secondary antibody: 1:400 Alexa Fluor 488 (Cat. No. A11001) from Molecular Probes, Life Technologies, or 1:400 daylight 549 for MAP2 (Cat. No. ab96948) from Abcam (Abcam, Cambridge, UK). Finally, cells were washed in PBS, incubated for 5 min with Hoechst 33342 from Sigma Chemical Co (St. Louis, MO, USA; Cat. No. H1399) (15 mg/L in PBS, pH 7.4) in the dark, and visualized by confocal microscopy. Images were acquired on a Zeiss LSM 710 (40 × 1.4NA plan-apochromat oil immersion lens) and analyzed using ImageJ software. An ImageJ macro tool was used to analyze mitochondrial network as previously described [38].

### Immunoblotting

To prepare cytosolic samples for phospho-Tau, Aβ oligomers, pro-IL-1β, TLR3, TLR4, NLRP3, and IkBα western blot analyses, after incubations, neurons were washed in ice-cold PBS and lysed in lysis buffer (10 mM HEPES; 3 mM MgCl_2_; 1 mM EGTA; 10 mM NaCl, pH 7.5, supplemented with 2 mM DTT, 0.1 mM PMSF and a 1:1000 dilution of a protease inhibitor cocktail) supplemented with 2 mM sodium orthovanadate and 50 mM sodium fluoride and 0.1% Triton X-100. Neurons were scraped on ice, transferred to the respective tubes and incubated on ice for 40 min. Afterwards, samples were centrifuged at 2300×*g* for 10 min at 4 °C and the resulting supernatant contains the cytosolic fraction. For preparation of nuclear extracts for the analysis of p65 NF-κB, the resulting pellets were suspended in lysis buffer (20 mM HEPES, 300 mM NaCl, 5 mM MgCl_2_, 0.2 mM EGTA, 20% glycerol, pH 7.5) supplemented with 2 mM DTT, 0.1 mM PMSF and a 1:1000 dilution of a protease inhibitor cocktail and samples were incubated on ice for 15 min. Subsequently, samples were centrifuged at 12000×*g* for 20 min at 4 °C and the resulting supernatant contains the nuclear fraction. To prepare samples for p62 protein level determination, the hypotonic lysis buffer (25 mM HEPES, pH 7.5, 2 mM MgCl_2_, 1 mM EDTA, and 1 mM EGTA supplemented with 2 mM DTT, 0.1 mM PMSF, and a 1:1000 dilution of a protease inhibitor cocktail) was supplemented with 1% Triton X-100. After 3 cycles of freezing (liquid nitrogen) and thawing (37 °C water bath), samples were centrifuged at 12000×*g* for 10 min and at 4 °C. The total amount of all resulting cell lysates obtained were removed and stored at − 80 °C. Protein content was determined using BCA Protein Assay protein assay (Pierce; Cat. No. 23227).

For the analysis of mitochondrial p-DRP1, neurons were washed with PBS, scraped, and disrupted on ice by homogenization in a buffer containing 250 mM sucrose, 20 mM Hepes, 1 mM EDTA, 1 mM EGTA, and protease inhibitors (0.1 M PMSF, 0.2 M DTT, 1:1000 dilution of a protease inhibitor cocktail and phosphatase inhibitors (1 tablet per 10 mL of lysis buffer) from Sigma Chemical Co (St. Louis, MO, USA; Cat. No. 04906845001). This suspension was centrifuged at 500×*g* for 12 min at 4 °C. The resulting supernatant was centrifuged at 9500×*g* for 20 min at 4 °C. Pellets resulting from this step contain a crude mitochondrial fraction. Mitochondrial pellets were resuspended in sucrose buffer and freeze (liquid nitrogen) -thawed (37 °C water bath) three times. The total amount of resulting mitochondrial fractions obtained were removed and stored at − 80 °C. For the analysis of Aβ oligomeric content mitochondrial samples were suspended in 2× sample buffer (40% glycerol, 2% SDS, 0.2 M Tris-HCl pH 6.8, 0.005% Coomassie Blue) and were not boiled (non-denaturing) and loaded under non-reducing conditions (in the absence of DTT in the sample buffer). For the analysis of the remaining proteins, samples were suspended in 6× sample buffer and boiled for 5 min at 95 °C (4× Tris.HCl/SDS, pH 6.8, 30% glycerol, 10% SDS, 0.6 M DTT, 0.012% bromophenol blue). Depending on the protein molecular weight of interest, samples containing 35 μg of protein were loaded onto 10% SDS-PAGE gels. Specifically, for the analysis of Aβ oligomers, samples were separated by electrophoresis on a 4–16% Tris–Tricine-SDS gel. After transfer to PVDF membranes (Millipore, Billerica, MA, USA), non-specific binding was blocked by gently agitating the membranes in 3% BSA and 0.1% Tween-20 in Tris-Buffered Solution (TBS—20 mM Tris, 150 mM NaCl, and 0.1% (w/v) Tween-20 ®, pH 7.6) for 1 h at room temperature. The blots were subsequently incubated with the respective primary antibodies overnight at 4 °C with gentle agitation: 1:1000 anti-p62 (Cat. No. P0067) from Sigma Chemical Co (St. Louis, MO, USA); 1:1000 p-Drp1 Serine 616 (Cat. No. 3455); 1:1000 anti- p65 NF-κB (Cat. No. 8242); 1:1000 anti-IκB-alpha (Cat. No. 4812) from Cell Signaling Technology (Cell Signaling, Danvers, MA, USA); 1:750 anti-NLRP3 (Cat. No. MA5-23919) from ThermoFisher Scientific (Waltham, MA, USA); 1:500 anti-Aβ (Cat.No. A5441) from BioLegend (San Diego, CA, USA); 1:1000 anti-TLR3 (Cat. No. ab84911) from Abcam (Abcam, Cambridge, UK); 1:500 anti-TLR4 (Cat. No. sc-293072), 1:1000 anti-IL-1β (Cat. No. sc-7884), and 1:750 anti-phospho-tau (p-tau) Thr181 (Cat. No. sc-101816) from Santa Cruz Biotechnology (Santa Cruz, CA, USA). Finally, 1:1000 anti-SDHA (Cat. No. ab137746), 1:1000 anti-TATA binding protein (Cat. No. MAB3658) from Merk Life Science SLU (from Sigma St. Louis, MO, USA), and 1:5000 anti-β-actin (Cat. No. A5441) were used for loading control. Membranes were washed with TBS containing 3% BSA and 0.1% Tween three times (each time for 5 min) and then incubated with the appropriate horseradish peroxidase-conjugated secondary antibody for 2 h at RT with gentle agitation. After three washes, specific bands of interest were detected by developing with an alkaline phosphatase enhanced chemical fluorescence reagent (ECF from Sigma St. Louis, MO, USA) (Cat. No. GERPN3685). Fluorescence signals were detected using a BioRad Versa-Doc Imager, and band densities were determined using Quantity One Software. Where indicated, protein densities were corrected (i.e., divided) with the densities of β-actin, TATA, or SDHA to account for possible differences in protein loading.

### IL-1β levels assessment by ELISA

After treatments, 200 μl of the extracellular neuronal media was collected in individual tubes. To pellet possible media contaminants, supernatant samples were centrifuged at 15000×*g* for 20 min at 4 °C. Neurons were then washed in ice-cold PBS and proteins extracted in lysis buffer I (10 mM HEPES; 3 mM MgCl_2_; 1 mM EGTA; 10 mM NaCl, pH 7.5) supplemented with 2 mM DTT, 0.1 mM PMSF, and a 1:1000 dilution of a protease inhibitor cocktail and 0.1% Triton X-100. After 40 min of incubation on ice, samples were centrifuged at 2300×*g*, 10 min at 4 °C. IL-1β levels were evaluated using mouse Quantikine ELISA (R&D Systems; Cat. No. MLB00C) according to the manufacturer’s instructions. Specifically, extracellular IL-1β levels were evaluated using 50 μl of each sample, whereas intracellular abundance of IL-1β was measured using 40 μg of protein for each condition.

### Caspase 1-like activity assay

Neuronal extracts were processed as described above except for the supplementation of Triton X-100. The resultant cell extracts were removed and stored at − 80 °C. Protein content was determined using BCA Protein Assay protein assay (Pierce).

Caspase 1 activation was measured using a colorimetric substrate in which the substrate cleavage was monitored at 405 nm. Cortical neurons lysates containing 50 μg protein were incubated at 37 °C for 2 h in 25 mM HEPES, pH 7.5 containing 0.1% CHAPS, 10% sucrose, 2 mM DTT, and 40 μM of Ac-VAD-4-methoxy-2-naphtylamide (Sigma Chemical Co (St. Louis, MO, USA; Cat. No. SCP0066) to determine caspase 1 activation. Detection was evaluated at 405 nm using a Spectramax Plus 384 spectrophotometer (Molecular Devices, Sunnyvale, CA, USA).

### Data analysis

Data are expressed as means ± standard error of the means (SEM) of at least 4 independent experiments. Statistical analyses were performed using GraphPad Prism 6 for Windows (GraphPad Software, San Diego, CA, USA). To compare means between groups, we used one-way analysis of variance (ANOVA) followed by Dunnett’s multiple comparisons (comparisons between untreated cells *versus* treatments). *p* values < 0.05 were considered significant.

## Results

### BMAA disrupts mitochondrial metabolism inducing fragmentation and cardiolipin exposure

**Fig. 1:**
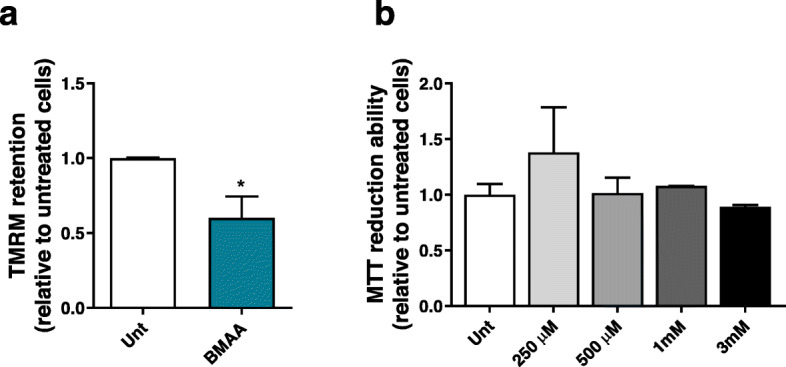
BMAA targets the mitochondria in cells lacking functional glutamate receptors. **a** SHSY-5Y cells, which do not express functional NMDA receptors, were exposed to 3 mM BMAA for 48 h. After this period, mitochondrial membrane potential was measured using TMRM^+^ dye. After baseline reading neurons were exposed to 1 μM of CCCP + 2.5 μM oligomycin to prevent reversal of ATP synthase. Bars depict maximum RFU after CCCP + oligomycin minus basal RFU reading. Data represent the mean ± SEM derived from six independent experiments and are expressed relative to untreated neurons. The statistical significance was determined using unpaired *t* test and *p < 0.05 when compared to untreated SHSY-5Y cells. **b** After 48 h of exposure to increasing concentrations of BMAA (250 μM; 500 μM; 1 mM and 3 mM), the MTT assay was performed to assess cell metabolic activity by measuring the formation of insoluble formazan. Data represent mean ± SEM values derived from three independent determinations and results are expressed relative to untreated neurons

It has been described that BMAA induces excitotoxicity in cultured neurons and cell lines through the activation of NMDA receptors [29], causing intracellular Ca^2+^ rise and mitochondrial-dependent ROS production [39], and to date, this was proposed as the main mechanism for BMAA-dependent neuronal death, followed by the misincorporation of BMAA into primary protein structure of protein through the replacement of serine [30, 40]. Here we show that in undifferentiated SHSY-5Y cells, that do not express functional NMDA receptors [41], 3 mM BMAA targets the mitochondria, inducing a significant reduction in mitochondrial membrane potential (ΔΨmit) (Fig. 1a) without significantly decreasing cell reducing capacity (Fig. 1b).

**Fig. 2:**
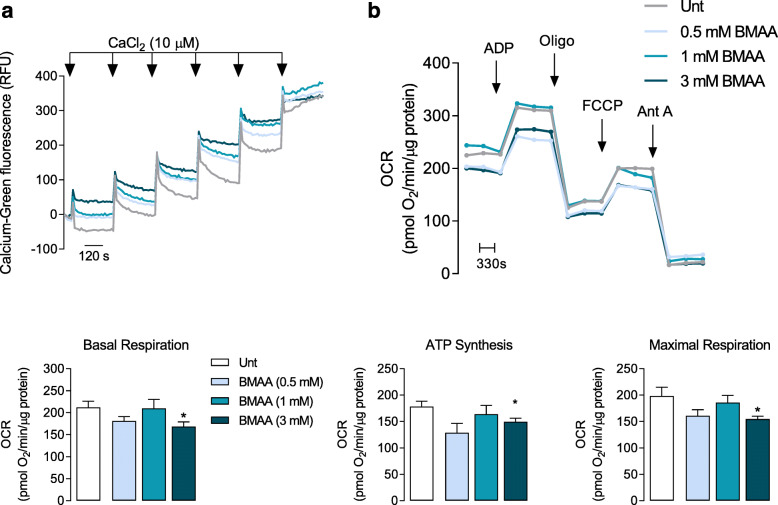
BMAA affects neuronal mitochondria oxygen consumption rate (OCR) and mitochondrial calcium uptake. The fundamental parameters of mitochondrial function namely ability to uptake calcium, basal respiration, ATP synthesis, and maximal respiration can be evaluated in isolated mitochondria. **a** Mitochondrial ability to uptake calcium was evaluated by Ca^2+^-sensitive fluorescent dye Calcium Green 5N, in isolated mitochondria treated or not with BMAA (0.5–3 mM), subjected to 10 μM Ca^2+^ pulses applied until the Ca^2+^ retention capacity was reached as shown by the upward deflection of the trace. **b** OCR representative curve and respective histograms of basal respiration, maximal respiration, and ATP synthesis were determined in isolated mitochondria from cortical tissue treated with different BMAA concentrations. The assay was performed in MAS containing 10 mM succinate plus 2 μM rotenone under sequentially injection of mitochondrial inhibitors and substrates (final concentration, 4 mM ADP; 2.5 μg/mL oligomycin; 4 μM FCCP and 4 μM antimycin A) as shown in representative traces using Seahorse XF24 extracellular flux analyzer. Data represent the mean ± SEM derived from six independent experiments and are expressed as pmol of O_2_/min/μg of isolated mitochondria. The statistical significance was determined using one-way ANOVA followed by Dunnett’s test *p < 0.05 versus untreated mitochondria

**Fig. 3:**
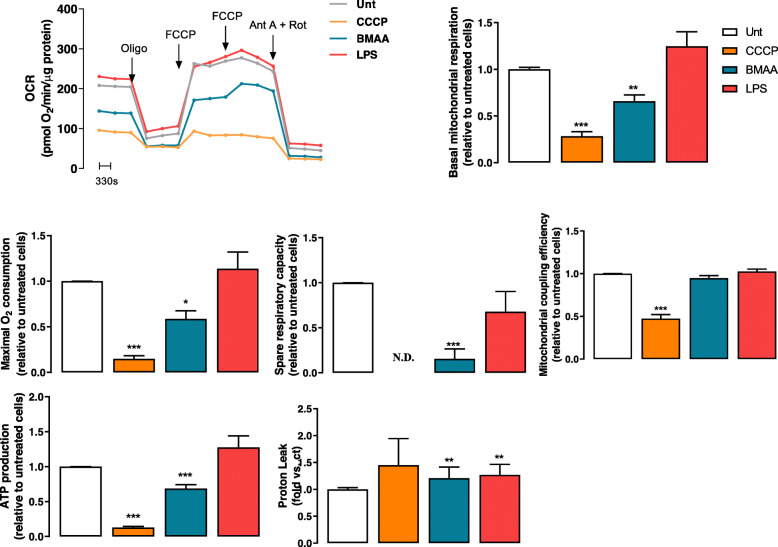
BMAA induces impairment of OCR in primary cortical neurons. OCR representative curve and respective histograms of Basal O_2_ consumption, maximal O_2_ consumption, coupling efficiency, ATP production, and proton leak were evaluated in primary mouse cortical neurons pre-treated with 1 μM CCCP, 3 mM BMAA, and 1 μg/μl LPS for 48 h, in the presence of 1 μM oligomycin, 2 μM FCCP, and 2 μM antimycin 2 μM plus rotenone. Incubations were performed in quadruplicate and data are the mean values of such experiments. Data represent the mean ± SEM derived from five independent experiments and are expressed relative to untreated cells. The statistical significance was determined using one-way ANOVA followed by Dunnett’s test *p < 0.05, **p < 0.01, and ***p < 0.001 *versus* untreated neurons. N.D., negligible OCR rates

**Fig. 4:**
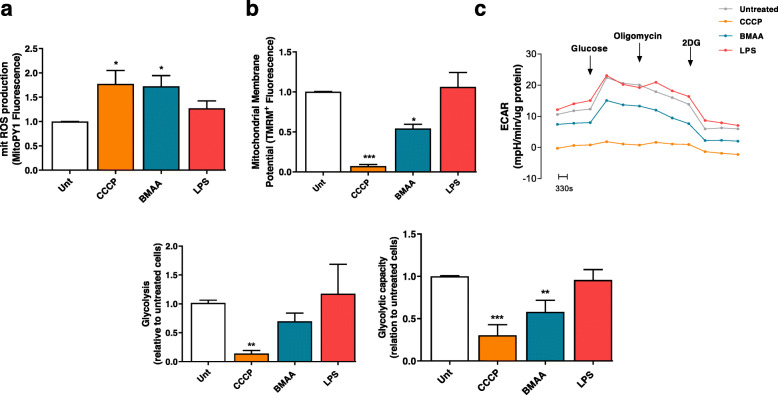
BMAA treatment increases mitROS, decreases ΔΨmit, and impairs neuronal glycolysis rates. In primary mouse cortical neurons pre-treated with 1 μM CCCP, 3 mM BMAA, and 1 μg/μl LPS for 48 h, we evaluated **a** mitochondrial ROS production using the fluorescent probe MitoPY1. MitoPY1 is selective for hydrogen peroxide of mitochondria in living cells. After baseline reading, neurons were exposed to 5 μM rotenone (mitochondrial complex I inhibitor). Bars depict maximum RFU after rotenone minus basal RFU reading. Data represent the mean ± SEM derived from six independent experiments and are expressed in relation to untreated neurons. The statistical significance was determined using one-way ANOVA followed by Dunnett’s test *p < 0.05 *versus* untreated neurons. **b** Mitochondrial membrane potential using the fluorescent cationic dye TMRM^+^. After baseline reading, neurons were exposed to 1 μM of CCCP plus 2.5 μM oligomycin to prevent reversal of ATP synthase. Bars depict maximum RFU after CCCP + oligomycin minus basal RFU reading. Data represent the mean ± SEM derived from six independent experiments and are expressed in relation to untreated neurons. The statistical significance was determined using one-way ANOVA followed by Dunnett’s test *p < 0.05 and ***p < 0.001 *versus* untreated neurons. **c** Extracellular acidification rate (ECAR) representative curve and respective histograms of glycolysis and glycolytic capacity were evaluated were evaluated in primary mouse cortical neurons pre-treated with 1 μM CCCP, 3 mM BMAA, and 1 μg/μl LPS for 48 h, using a Seahorse XF24 extracellular flux analyzer. Incubations were performed in quadruplicate and data are the mean values of such experiments. Data represent the mean ± SEM derived from four independent experiments and are expressed relative to untreated cells. The statistical significance was determined using one-way ANOVA followed by Dunnett’s test **p < 0.01 and ***p < 0.001 *versus* untreated neurons

**Fig. 5:**
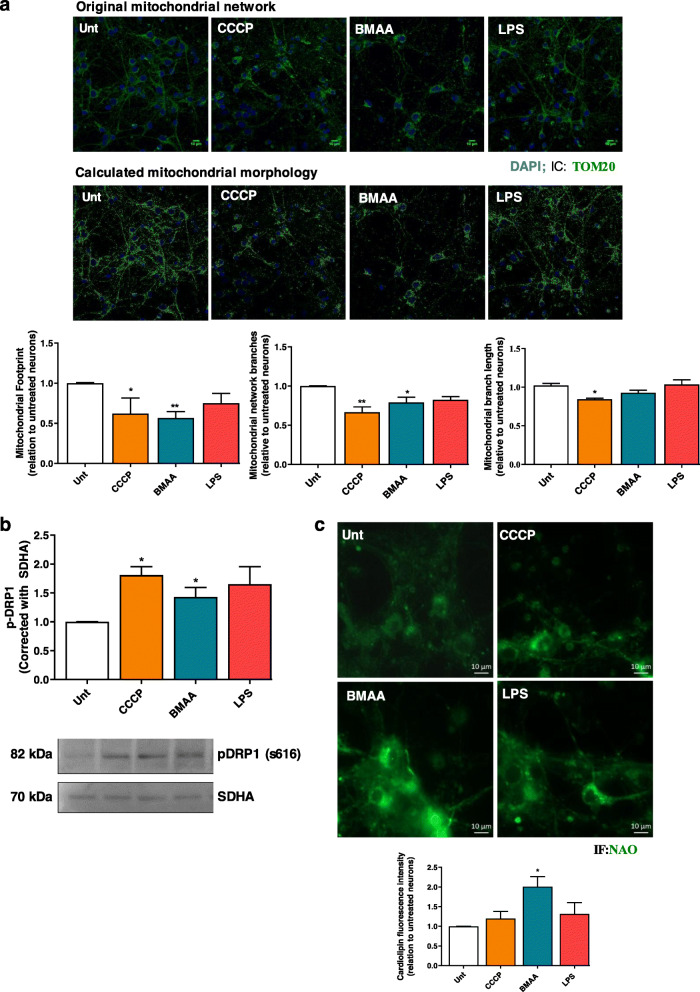
Bacterial effectors disrupt mitochondrial network and expose cardiolipin. In primary mouse cortical neurons pre-treated with 1 μM CCCP, 3 mM BMAA, and 1 μg/μl LPS for 48 h, we evaluated **a** representative images of mitochondrial network immunostained with TOM20. Respective histograms of mitochondrial footprint, mitochondrial network branches, and mitochondrial branch length were calculated using Mitochondrial Network Analysis (MiNA) toolset, a relatively simple pair of macros making use of existing ImageJ plug-ins, allowing for semi-automated analysis of mitochondrial networks in cultured mammalian cells. MiNA is freely available at https://github.com/ScienceToolkit/MiNA. This MiNA toolset allows the analysis and quantification of various parameters of mitochondrial network namely mitochondrial footprint (distribution), network branching, and branch length. The panel corresponding to the calculated mitochondrial morphology shows mitochondrial network without the noise that characterizes fluorescent images. This filter is applied to a specified radius of two pixels and the pixel value is changed to the median of the surrounding pixels of that radius, removing noise from the image, allowing a clearer view of mitochondrial footprint. Values are mean ± SEM of four independent experiments and are expressed relative to untreated neurons. The statistical significance was determined using one-way ANOVA followed by Dunnett’s test *p < 0.05, **p < 0.01 versus untreated neurons. **b** Mitochondrial fission by detecting pDrp1 s616 fission protein by western blotting. Representative immunoblot and quantification analysis of pDrp1 s616 protein levels was performed. Equal protein amounts were loaded and confirmed with succinate dehydrogenase complex, subunit A (SDHA) staining. Data represent mean ± SEM values derived from four independent determinations. The statistical significance was determined using one-way ANOVA followed by Dunnett’s test *p < 0.05, when compared to untreated neurons and **c** cardiolipin exposure using the fluorescent dye 10-*N*-nonyl acridine orange (NAO), which is extensively used for location and quantitative assays of cardiolipin in living cells. Cardiolipin fluorescence intensity was calculated with ImageJ. Values are mean ± SEM of four independent experiments and are expressed relative to untreated neurons. The statistical significance was determined using one-way ANOVA followed by Dunnett’s test *p < 0.05, **p < 0.01 *versus* untreated neurons

In our work, we challenged isolated cortical mitochondria from C57BL/6 mice with increasing doses of BMAA (0.5–3 mM). Mitochondrial Ca^2+^ uptake capacity was monitored through Calcium Green-5N fluorescence in mitochondrial suspensions subjected to 10 μM Ca^2+^. Our results provide evidence that a single pulse of 10 μM Ca^2+^ induced a rapid Ca^2+^ uptake by isolated mitochondria and that the kinetics decreased following BMAA treatment (Fig. 2a). In this cell-free system, 3 mM BMAA also decreased mitochondrial O_2_ consumption namely basal and maximal respiration with concomitant decrease in ATP production (Fig. 2b), which shows that BMAA exerts a direct effect on mitochondrial function. Primary cortical neurons were then exposed to 3 mM BMAA, 1 μg/μl LPS (an endotoxin found in the outer membrane of Gram-negative bacteria that acts as a pathogen-associated molecular pattern-PAMP), and 1 μM CCCP (mitochondrial uncoupler) as described in the “Methods” section. Oxygen consumption rates (OCR) were significantly decreased in CCCP and BMAA-treated neurons for all parameters evaluated with exception of proton leakage (Fig. 3). LPS altered some parameter, but its effect on OCR was not conclusive. BMAA effect on ORC is in accordance with the increased levels of mitochondrial ROS produced (Fig. 4a) and with the decreased ΔΨmit (Fig. 4b). Since glycolysis could be upregulated as a direct neuronal response to increased energy demands [42], we determined extracellular acidification rates (ECAR) and observed a reduced glycolytic capacity of neurons exposed to BMAA or CCCP (Fig. 4c). Mitochondrial dysfunction is usually followed by organelle fragmentation in order to allow the degradation of the less efficient components by mitophagy. Structurally, neuronal mitochondrial network, as visualized with TOM20 staining, was fragmented upon BMAA and CCCP exposure (Fig. 5a), which correlates with increased mitochondrial phospho-DRP1 at serine 616 (Fig. 5b). A fragmented mitochondrial network may expose components from the inner mitochondrial membrane that are usually inaccessible. Using NAO dye for specific staining of cardiolipin, neurons treated with BMAA showed significant increase in cardiolipin fluorescence (Fig. 5c). Cardiolipin, a DAMP, can bind to the leucin-rich LRR domain of NLRP3 inflammasome leading to its activation [43], but it can also recruit the autophagic machinery, interacting with LC3 and promote mitophagy [44].

### BMAA activates neuronal innate immunity through mitochondria

Although innate immunity alterations are intimately linked to AD and other neurodegenerative disorders, it remains unclear why innate immunity is altered in the disease state and whether changes in immunity are a cause or a consequence of neuronal dysfunction [45]. Additionally, it has been described that microglial cells are responsible for the neuroinflammatory events that occur in AD neurodegenerative process. In order to discriminate the effect of BMAA in neurons, primary cell cultures were treated with FDU [46] to obtain pure neuronal cultures (virtually undetected glia cells). A control with no added FDU was performed revealing where the expected presence of astrocytes was determined with GFAP staining (Fig. S1a1). Additionally, we did not see Iba1-positive microglial cells in our preparation (Fig. S1a2). In cells treated with FDU, there is no detectable GFAP, which resulted in pure neuronal cultures with exclusive MAP2 staining (Fig. S1a3).

**Fig. 6:**
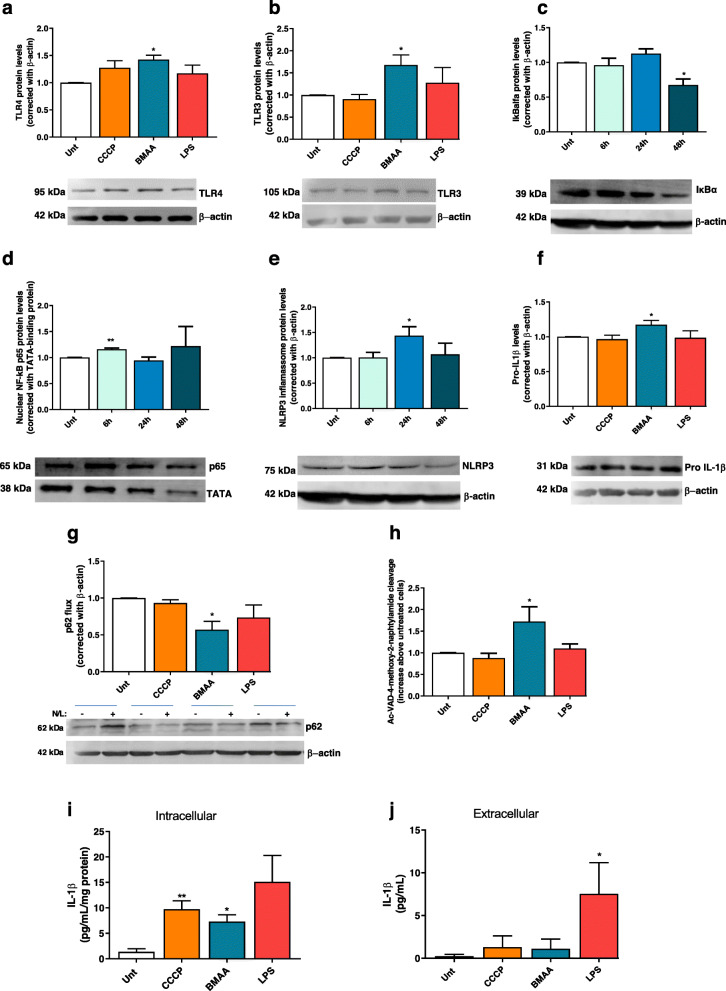
BMAA activates neuronal innate immunity. In primary mouse cortical neurons pre-treated with 1 μM CCCP, 3 mM BMAA, and 1 μg/μl LPS for 48 h or with 3 mM BMAA for6, 24 and 48 h, we evaluated **a** changes in protein levels of TLR4 and **b** TLR 3 by western blotting. Representative immunoblot and densitometric analysis was performed by the loading of equal amounts of protein corrected with β-actin. Inflammasome activation was determined by the quantification of **c** cytosolic IkBα protein levels which were determined by western blotting. Representative immunoblot and densitometric analysis was performed by loading equal amounts of protein corrected with β-actin. **d** p65 NFκB was detected in enriched nuclear fractions by western blotting. Representative immunoblot and densitometric analysis was performed by the loading of equal amounts of protein corrected with TATA-binding protein. **e** Cytosolic NLRP3 protein levels were evaluated by western blotting. Representative immunoblot and densitometric analysis was performed by loading equal amounts of protein corrected against β-actin. **f** Cytosolic pro-IL-1β protein levels analyzed by western blotting. Representative immunoblot and densitometric analysis was performed by the loading of equal amounts of protein corrected with β-actin. **g** p62 flux was evaluated by western blotting after 4-h incubation with or without NH_4_Cl plus leupeptin. Representative immunoblot and densitometric analysis was performed by the loading of equal amounts of protein corrected with β-actin. **h** Caspase 1-like activity was determined by spectrophotometry at 450 nm, and **i** quantification of IL-1β levels in cytosolic fractions and **j** in supernatants was determined using ELISA. Data represent mean ± SEM values derived from four to six independent determinations and are expressed relative to untreated neurons. The statistical significance was determined using one-way ANOVA followed by Dunnett’s test *p < 0.05 and **p < 0.01 when compared to untreated neurons

In pure cortical neurons exposed to BMAA, there is an increase in the expression of the cell surface TLR4 and in the intracellular endosome associated TLR3 (Fig. 6a, b). TLR3 localizes preferentially within neuronal growth cones and its activation has been linked to impaired axonal development, constituting a neuronal mechanism for damage sensing [47, 48]. In response to LPS, IkB kinase (IKK) is activated to phosphorylate the N-terminal of NF-κB-IkBα complex leading to IkB subsequent ubiquitination and degradation by the proteasome. NF-κB dimers translocate to the nucleus to activate the transcription of various target genes [49]. Our results show that 3 mM BMAA treatment decreases IkBα cytosolic levels (Fig. 6c), allowing the translocation of p65 NF-κB to the nucleus (Fig. 6d). Here, NF-κB transcription factor activates the transcription of NLRP3 components and pro-IL-1β (Fig. 6e,f). Recently, p62 involvement in the mechanism that allows NF-κB to regulate NLRP3 inflammasome activation and cytokine release in macrophages was revealed [50]. NF-κB stimulates p62 transcription to allow the recognition of damaged mitochondria and promote their clearance by mitophagy [51]. In neurodegenerative diseases such as AD, the accumulation of dysfunctional mitochondria occurs due to defects on macroautophagy [10, 52], which results in p62 accumulation that will amplify NF-κB-dependent inflammation [50]. Our results show that, upon BMAA treatment, p62 flux is impaired (Fig. 6g) favoring NLRP3-dependent activation of caspase 1 and subsequent cleavage of pro-IL-1β into its mature form and release to the extracellular medium (Fig. 6h–j). Furthermore, we observed that LPS can sensitize neurons to release IL-1β, but this regulation seems independent of mitochondria and of NF-κB activation (Fig. 6j).

### Aβ oligomers and Tau phosphorylation increase after exposure to bacterial metabolites

**Fig. 7:**
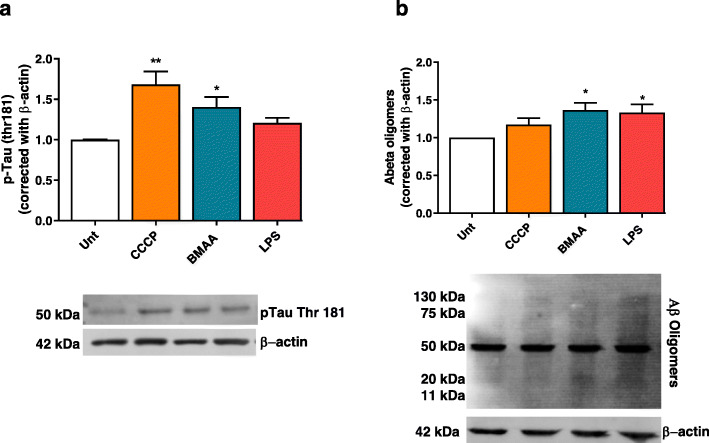
Bacterial effectors induce AD pathological hallmarks in cortical neurons. In primary mouse cortical neurons pre-treated with 1 μM CCCP, 3 mM BMAA, and 1 μg/μl LPS for 48 h, we determined **a** p-Tau Thr181 protein levels by western blot. Representative immunoblot and densitometric analysis was performed by the loading of equal amounts of protein corrected with β-actin. Data represent mean ± SEM values derived from six independent determinations and data are expressed relative to untreated neurons. *p < 0.05, when compared to untreated neurons and **b** cytosolic Aβ oligomers protein levels by PAGE. Representative immunoblot and densitometric analysis was performed by the loading of equal amounts of protein corrected with β-actin. Data represent mean ± SEM values derived from six independent determinations and data are expressed relative to untreated neurons. The statistical significance was determined using one-way ANOVA followed by Dunnett’s test *p < 0.05, when compared to untreated neurons

Neuroinflammation is a key process during AD that can modulate the processing of Aβ peptides and the increase in Tau phosphorylation, the major AD neuropathological hallmarks, and thus modulate disease pathogenesis [53]. Recently, it was demonstrated that by regulating NLRP3 activation, it was possible to decrease the release of pro-inflammatory factors by brain microglia, which contributed to a reduced phospho-p38 and tau hyperphosphorylation in SHSY-5Y cells [54]. Our results show that both CCCP and BMAA mediate the phosphorylation of Tau at Thr181 probably through mitochondrial metabolism failure in neurons (Fig. 7a). Regarding Aβ oligomers production, evidence support that the increased production of neuroinflammatory cytokines results in increased APP expression, which may be linked to increased Aβ processing and further deposition [55]. Our results point to an increase of intracellular Aβ oligomers, with most significance after treatment with BMAA, or exposure to LPS (Fig. 7b).

## Discussion

The mitochondrial cascade hypothesis for AD postulates that mitochondrial dysfunction precedes APP processing into Aβ, Tau phosphorylation, and neuroinflammation, all distinctive hallmarks of the disease [12, 56]. Interestingly, mitochondria are evolutionary symbionts of early eukaryotic cells, having their origin in a proteobacterial lineage [57], which makes them natural targets of many microbial byproducts. Recently, a novel class of non-photosynthetic cyanobacteria designated Melainabacteria was identified in tap and groundwater as well as in the human gut [58]. This newly identified group of bacteria may be a candidate for BMAA production within the gut, as a putative strategy to cope with the competitive pressure in such an overpopulated environment, a mechanism that was probably retained during evolution [59]. Although BMAA has been characterized as a non-proteinogenic amino acid that can be misincorporated into proteins [30] causing endoplasmic reticulum stress, redox imbalance, and caspase-dependent apoptotic cell death [31], we present evidence for its direct action on mitochondria. Several microbial substances are known to specifically target mitochondria and induce neuronal death, namely pneumolysin from *Streptococcus pneumoniae*, a pore-forming toxin [60]. Other cytotoxic microbial effectors causing mitochondrial impairment are the *Staphylococcus aureus* α-toxin and the vacuolating cytotoxin (VacA) from *Helicobacter pylori* [61]. Interestingly, BMAA elicited a pronounced decrease in oxidative phosphorylation, impaired calcium homeostasis, and exacerbated ROS production in cells of the NSC-34 neuronal cell line [62]. In addition, BMAA also dramatically affected the viability of N2a neuronal cell line, inferred from the drop in the activity of the mitochondrial succinate dehydrogenase (SD) [63]. The common origin of mitochondria and bacteria may facilitate the direct import of certain bacterial metabolites into mitochondria. A plausible route for bacterial toxins to reach mitochondria is their incorporation into endosomal vesicles trafficking and the consequent release into the cytosol before entering the mitochondria [64]. This is the delivery pathway of toxins from *Clostridium difficile*, which are released into the cytoplasm by pore formation on the lysosome wall and, subsequently, localize at the mitochondria where they induce apoptosis [65]. Further work has provided evidence that bacterial endotoxins, such as LPS, cause mitochondrial dysfunction while causing neuroinflammation in specific brain regions. Wild-type animals subjected to systemic injection of LPS showed a higher concentration of cytokines and microglia activation in the frontal cortex, which was also elevated in other areas such as the striatum and the hippocampus [66]. It was previously shown in AD models that mitochondrial network is highly fragmented and that mitochondrial fission is essential to selectively target dysfunctional mitochondria for degradation by mitophagy [10]. Also in agreement with our results, the resulting exposure of the inner membrane phospholipid cardiolipin serves an important defensive function in eliminating damaged mitochondria [44]. Since cardiolipin is found only in the inner mitochondrial membrane and in the membranes of most bacteria, this lipid is considered a mitochondrial-derived DAMP recognized by NLRP3 [67]. Since neurons have higher susceptibility to mitochondrial toxins due to the metabolic shift from glycolysis to OXPHOS during neuronal differentiation [68], we propose that neurons are sentinels of brain insults. Our results also reveal that neurons sense and respond to BMAA with the production of cytokines and possibly other inflammatory cues that will further activate glial cells, resulting in astrogliosis, a common histopathological hallmark of AD. A significant number of studies have shown that neurons have the machinery to respond to pathogens through activation of neuronal innate immunity. Also in support of our results that show neuronal increase in TLR3 and 4, evidence from more than a decade ago had shown that human neuronal cells express TLR3 and TLR8 and can mount innate immunity responses against double-stranded RNA Rabies virus and single-stranded RNA herpes simplex virus [69, 70]. Neurons were found to express other TLRs [71], namely TLR4 which activation upon Aβ_1-42_ exposure was related with enhanced caspase activation and neuronal death [72]. In agreement with our observations, these neurons also produced chemokines, inflammatory cytokines such as IL-6, and interferon (IFN) that may mediate innate immunity in the absence of glial cells. Recent evidence further demonstrated that neurons alone can activate NF-κB-dependent NLRP3 inflammasome in response to ischemic insults [73, 74]. We extend these insightful results by reporting here that some bacterial metabolites can indeed activate neuronal NLRP3 inflammasome in pure cortical neuronal cultures. Thus, the controversy about the role of Aβ peptides in AD progression may be reconciled with the evidence that NLR and TLR activation also trigger the production of pro-inflammatory cytokines and antimicrobial peptides (AMPs) [75]. A previous study in monkeys showed that dietary BMAA can prompt the formation of neurofibrillary tangles and Aβ plaques characteristic of ALS-PDC and AD [76]. Accordingly, we show that after exposure to BMAA and LPS, neurons have increased Aβ oligomers content, demonstrating the potential of neurons alone to fight bacterial infections. Recently, it was demonstrated that Aβ reduces microbial adhesion to host cells and agglutinates and entraps microbes in fibrils [77]. VHHQK2 heparin-binding domain of Aβ binds microbial cell wall carbohydrates, which work as seeds for Aβ fibrils propagation. Interestingly, Aβ peptides are resistant to bacterial proteases, being able to induce pores in bacterial membranes, thus blocking the life cycle of pathogens [77]. Regarding Tau phosphorylation, recent data pointed out that aggregated Tau activates NLRP3 through NLRP3-adaptor protein ASC inflammasome [78] having an important role in regulating inflammation during disease progression. Also, it was demonstrated that by regulating NLRP3 activation it is possible to decrease the release of pro-inflammatory factors by brain microglia which reduced phospho-p38 and tau hyperphosphorylation in SHSY-5Y cells [54]. Altogether, the results presented here support our arguments in favor of a direct effect of a microbial neurotoxin in mitochondria, hence in the cascade of events that lead to AD neurodegeneration.

## Conclusions

Herein, we present data that supports our hypothesis that the bacterial-derived toxin BMAA targets the mitochondria, through mechanisms that might result from a number of evolutionary determinants shared between bacteria and mitochondria. By revealing a direct link between BMAA-induced mitochondrial dysfunction and neurodegeneration, including the development of AD features, such as Aβ peptides oligomerization and Tau hyperphosphorylation, along with the demonstration that cortical neurons are indeed capable of activating the NLRP3 inflammasome and of producing mature IL-1β, we here define a novel plausible pathway to sporadic AD. Future research is warranted to address if and how dietary and gut microbiome toxins, gut dysbiosis, and gut inflammation may collectively contribute to increased gut and BBB permeability and how these phenomena may impact the brain toward AD-related neurodegeneration.

## Supplementary Information

**Additional file 1:** **Supplementary Figure 1.** Effect of FDU treatment in cortical neurons enrichment. Immunostaining of GFAP, Iba1 and MAP2 in cortical neurons without FDU treatment **(a1-a2)** and FDU treated cortical neurons **(a3)**.

## Data Availability

The datasets used and/or analyzed during the current study are available from the corresponding author on reasonable request.

## Abbreviations

AD: Alzheimer’s disease
ALS-PDC: Amyotrophic lateral sclerosis-Parkinsonism dementia complex
AMPs: Antimicrobial peptides
BBB: Blood-brain barrier
BMAA: β-*N*-Methylamino-l-alanine
CCCP: Carbonyl cyanide m-chlorophenyl hydrazone
CNS: Central nervous system
CSF: Cerebrospinal fluid
DAMPs: Danger-associated molecular patterns
ECAR: Extracellular acidification rate
ENS: Enteric nervous system
FDU: 5-Fluoro-2’-deoxyuridine
LPS: Lipopolysaccharides
MTT: 3-(4,5-Dimethylthiazol-2-yl) 2,5-diphenyltetrazolium bromide
NAO: 10-*N*-Nonyl acridine orange
NH_4_Cl: Ammonium chloride
NLR: Nod-like receptor
OCR: Oxygen consumption rate
sAD: Sporadic Alzheimer’s disease
TLR: Toll-like receptor
TMRM: Tetramethylrhodamine methyl ester
