# Supplementary figures and images for: Microbial BMAA elicits mitochondrial dysfunction, innate immunity activation, and Alzheimer’s disease features in cortical neurons
Source: J Neuroinflammation. 2020 Nov 5;17:332. doi: 10.1186/s12974-020-02004-y (PMC7643281; doi:10.1186/s12974-020-02004-y)

## Slide 1
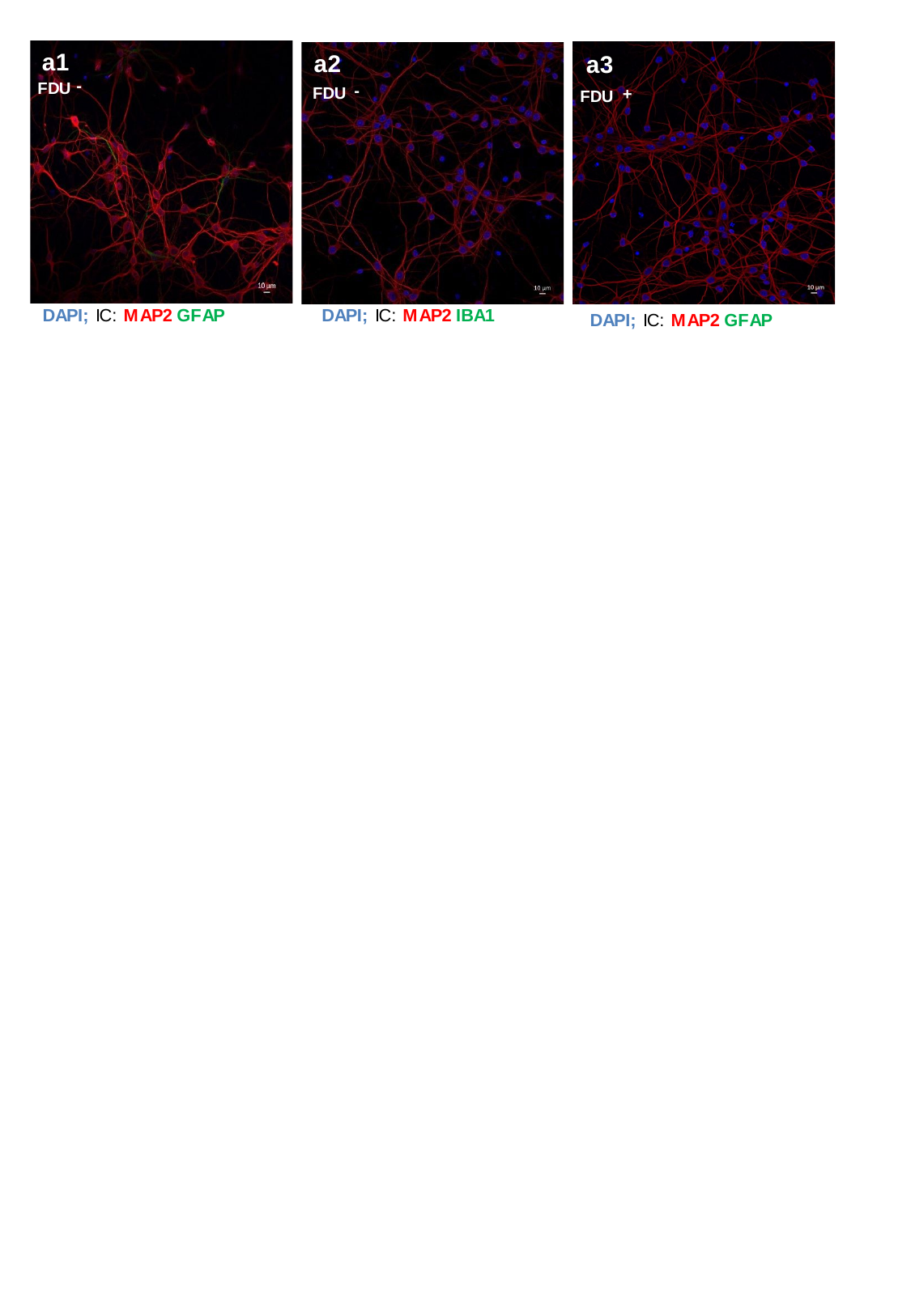

Supplement: Supplementary file 1 — Additional file 1: Supplementary Figure 1. Effect of FDU treatment in cortical neurons enrichment. Immunostaining of GFAP, Iba1 and MAP2 in cortical neurons without FDU treatment (a1-a2) and FDU treated cortical neurons (a3). [file 12974_2020_2004_MOESM1_ESM.pptx]
